# Standardizing clinical culture specimen collection in Ethiopia: a training‐of‐trainers

**DOI:** 10.1186/s12909-021-02631-w

**Published:** 2021-04-07

**Authors:** Jennifer Kue, Ashley Bersani, Kurt Stevenson, Getnet Yimer, Shu-Hua Wang, Wondwossen Gebreyes, Carmen Hazim, Matthew Westercamp, Michael Omondi, Berhanu Amare, Gebrie Alebachew, Rajiha Abubeker, Surafel Fentaw, Eyasu Tigabu, Denise Kirley, Daniel Vanderende, Elizabeth Bancroft, Kathleen M. Gallagher, Theresa Kanter, Joan-Miquel Balada-Llasat

**Affiliations:** 1 College of Nursing, The Ohio State University, Columbus, OH USA; 2 The Ohio State University Global One Health initiative, Columbus, OH USA; 3 The Ohio State University Wexner Medical Center, Columbus, OH USA; 4 U.S. Centers for Disease Control and Prevention, Atlanta, GA USA; 5 U.S. Centers for Disease Control and Prevention, Addis Ababa, Ethiopia; 6 Ethiopian Public Health Institute, Addis Ababa, Ethiopia

**Keywords:** Specimen collection, Training‐of‐trainers, Healthcare personnel, Ethiopia

## Abstract

**Background:**

Proper specimen collection is central to improving patient care by ensuring optimal yield of diagnostic tests, guiding appropriate management, and targeting treatment. The purpose of this article is to describe the development and implementation of a training-of-trainers educational program designed to improve clinical culture specimen collection among healthcare personnel (HCP) in Ethiopia.

**Methods:**

A Clinical Specimen Collection training package was created consisting of a Trainer’s Manual, Reference Manual, Assessment Tools, Step-by-Step Instruction Guides (i.e., job aides), and Core Module PowerPoint Slides.

**Results:**

A two-day course was used in training 16 master trainers and 47 facility-based trainers responsible for cascading trainings on clinical specimen collection to HCP at the pre-service, in-service, or national-levels. The Clinical Specimen Collection Package is offered online via The Ohio State University’s CANVAS online platform.

**Conclusions:**

The training-of-trainers approach may be an effective model for development of enhanced specimen collection practices in low-resource countries.

## Background

The use of proper procedures to collect clinical specimens for microbiology culture, mainly bacteria, mycobacteria, and fungi, is essential for accurate disease diagnosis and treatment. Standardizing the procedures that front-line healthcare personnel (HCP), such as nurses, physicians, and laboratory medical technologists/technicians with responsibilities that include direct patient contact, use to conduct and teach collection of specimens is critical for disease control and prevention[1]. Yet, standardized specimen collection procedures are lacking in many low- and middle-income countries (LMIC), such as Ethiopia[2]. One study conducted in three government run hospitals in Ethiopia reported that just 64.2 % of HCP received training on how to collect blood samples in college; while only 9.2 % received on-the-job training[3].

Erroneous microbiology culture results may range from false negative results due to improper storage conditions and microbial death to false positive results due to collection of normal flora rather than pathogenic microbes. These results can affect patient care as well as hospital infection control, patients’ length of stay, associated costs, patient safety, laboratory efficiency, and ultimately patient outcomes[1, 3, 4]. In order for laboratory results to be reliable, the pre-analytical phase of microbiology testing, which involves the collection, storage, transportation, and processing of specimen, must be conducted under strictly controlled procedures. Improved specimen collection can enhance healthcare delivery. As such, the Ethiopian Public Health Institute (EPHI) in collaboration with the United States Centers for Disease Control and Prevention (CDC), the American Society for Microbiology (ASM), and The Ohio State University Global One Health initiative (OSU GOHi) developed the Ethiopian Antimicrobial Resistance (AMR) Surveillance Plan in April 2017[4]. One of the main objectives of the plan was to establish a surveillance network capable of detecting priority AMR pathogens, analyzing and reporting susceptibility data, characterizing resistance by additional studies of bacterial isolates at EPHI laboratory, and generating evidence to inform the implementation of targeted prevention and control programs[4]. The plan outlines the activities needed to implement AMR surveillance, the approach for data collection, management, and reporting, and the roles and responsibilities of clinical laboratory stakeholders.

To ensure the quality of data collected for clinical care and AMR surveillance, OSU GOHi prioritized activities to focus on the pre-analytical stages of microbiology, namely ensuring quality of clinical specimen collection. We developed an integrated clinical specimen collection training package and implementation plan for local ministerial, academic, and hospital partners to ultimately target HCP, such as physicians, nurses, and laboratorians, who are responsible for collecting specimens for microbiology cultures.

The purpose of this paper is to provide an overview of the process of developing a standardized and sustainable specimen collection training package, from the initial needs assessment to its implementation, and the lessons learned from the training-of-trainers (ToT) cascade, which may serve as a model for other low- and middle -income countries. The goals of the training package and accompanying trainings included: (1) outlining the need for proper specimen collection to optimize treatment for patients with infectious diseases; (2) training participants on the standard and basic methods to obtain blood, wound, urine, respiratory, and stool samples for culture; and (3) providing basic information on skills needed to protect HCP and patients from transmission of infectious agents (e.g., proper hand hygiene, proper handling, disposal, and transport).

## Methods

### Needs Assessment

Informal needs assessments conducted at select healthcare facilities by OSU and EPHI staff in December 2016, highlighted the need for targeted efforts to improve the quality of specimens submitted for microbiology culture. In August 2017, OSU GOHi used its One Health Summer Institute as a platform to gather additional information on HCP training needs and the challenges in ensuring proper specimen collection in the Ethiopian context. OSU and EPHI staff held a workshop with key stakeholders in attendance, including physicians, nurses, laboratorians, and microbiologists from the sites participating in Ethiopia’s AMR Surveillance Network. The workshop provided participants with an overview of collection, transportation, and processing of clinical specimens and engaged them in discussions on challenges of sample collection, aseptic technique, transport, and biosafety. The workshop utilized each participant’s past knowledge and experience while providing new knowledge and building competencies. OSU staff obtained feedback from all stakeholders involved, including information on local resources and the local context, which helped to contextualize the training materials to be developed. Out of this discussion, OSU staff obtained information on the gaps in knowledge, skills, and laboratory capacity related to clinical specimen collection. Participant feedback and lessons learned from the workshop were incorporated into the development of the clinical specimen collection training package (described below).

### Material Development

The clinical specimen collection training package was designed to provide general information on specimen collection with focus on five commonly collected types of specimens for bacteria, mycobacteria and fungi cultures in Ethiopia: blood, wounds (skin and soft tissue), urine, respiratory secretions, and stool. Development and refinement of the training package was an iterative process, which involved modifying materials based on stakeholder feedback and after each round of trainings. Both master trainer and facility trainers received flash drives with copies of draft materials during the ToT to provide easy access for comments and feedback, which further informed refinement of the materials. Technological availability and dependability were also major considerations during the creation of the training package. The training materials were designed to be in a user-friendly, flexible format using adult learning principles[6]. The training package (described below) included a trainer’s manual, reference manual, assessment tools, core module PowerPoint slides, and step-by-step Reference guides, referred to as job aides.

#### 1. Trainer’s Manual

The Trainer’s Manual was designed for use with the ToT course on Clinical Specimen Collection. It contains the following: instructions on how to use the training materials, guidance on the theories of adult learning, how to best teach learners at different levels, a recommended course agenda for the training, core modules that focus on each specimen collection type, and an assessment tool for participants. There are six core training modules. The first module provides a general overview of good practices for specimen collection, transport, and timely processing to reduce contamination and ensure the sample is viable upon testing. The remaining five modules cover culture collection of the five aforementioned specimen types (see Table 1). While the materials for this exercise were tailored specifically for the Ethiopian context, generic open-source training materials will be available online: https://www.canvas.net/browse/osu/global-one-health.

**Table 1:** Specimen collection training module description

Module	Topics Covered
Module 1: Specimen Collection, Transport, and Processing	• General Concepts and Overview of Specimen Collection
	• Good Clinical Specimens
	• Keeping Specimens Free of Contamination
	• Collection Specimens at Right (Optimal) Time
	• Collecting Specimens in the Right Container
	• Collecting the Right Amount
	• Ensuring Specimens are Correctly Labeled
	• Safety and Transport
	• Rejection Criteria and Disposal
Module 2: Blood Culture Collection	• Blood Stream Infections
	• Blood Cultures and Methods
	• Blood Culture Collection from Peripheral Vein
	• Blood Culture Collection from Central Venous Catheter
Module 3: Wound (Skin and Soft Tissue) Culture Collection	• Wound Cultures
	• Collection Methods
	• Collection Procedures
Module 4: Urine Culture Collection	• Urine Cultures
	• Contamination Prevention
	• Types of Collection:
	Female
	Male
	Indwelling catheters
	Pediatric Patients
Module 5: Respiratory Culture Collection	• Respiratory Cultures
	• Types of Collection:
	Nares
	Nasopharyngeal
	Throat
	Lower Respiratory
Module 6: Stool Culture Collection	• Overview of Stool and Gastrointestinal Tract Cultures
	• Stool Collection Kit
	• Stool Cultures and Collection

#### 2. Reference Manual

The Reference Manual is an abridged version of the Trainer’s Manual and contains reference and instructional material for front-line HCP. The manual contains plain language, figures, and was translated to Amharic, the local language to allow for on-the-job training (group-based or self-study) of front-line HCP responsible for collection of clinical specimens.

#### 3. Assessment tools and checklists for trainers and participants

These materials assess understanding of the training sessions, material content, and training evaluation. Each assessment, consisting of questionnaires with scoring, were administered before the training and following the completion of each module. Competency checklists were developed to monitor improvement as competency assures knowledge uptake or skill acquisition. Directions were provided on how to administer the assessments in each module.

#### 4. Core module PowerPoint slides

Presentation slides for the six modules were provided, both as hard copies as well as electronically, to be used as adjunct and supplementary to the training sessions.

#### 5. Step-by-Step reference guides

Reference guides (i.e., job aides) for each specimen collection module with clear, concise, written steps on respective culture collection and supplemental illustrations, were developed and translated into Amharic. These were to be used by front-line HCP for quick review at the bed-side or for posting in the nurses stations as a consistent tool for use with each specimen collection.

#### 6. Course preparation materials

The Trainer’s Manual includes a list of materials, equipment, and supplies for facilitating the training. Also included was a list of preparations for trainers to consider prior to the start of the course, such as an agenda, registration forms, setting up the classroom, and providing training materials and supplies.

### Training Roll out

The roll out of the training package employed the CDC’s ToT model, which is intended to engage master trainers at the national level in coaching new trainers from various healthcare facilities[5]. The vision for Ethiopia was to develop a cadre of master trainers that could hold annual or semi-annual training at the national level (central location) to build a larger pool of competent instructors, or “facility-based trainers” who would then use the materials at their respective institutions and teach the materials to other front-line HCP.

Selection criteria for individuals to be considered for the role of master and facility-based trainers were developed by EPHI, CDC, and OSU program stakeholders. Individual level criteria included, but not limited to, having: (1) a bachelor’s degree in medical services (e.g., nursing, laboratory technologist, clinical microbiologist); (2) a minimum of one year in clinical practice (e.g., medical, laboratory, or nursing); (3) experience working in infectious diseases or other related field; (4) excellent communication and organizational skills; (5) high level of commitment and willingness in providing facility level trainings; (6) prescribed time to facilitate trainings at the facility level; and (7) be a permanent staff in the selected institution. Institutions also had to: (1) commit to participate in AMR surveillance; (2) have the microbiologic capability with enhanced specimen collection and patient data capture; (3) have an adequate client volume; and (4) have commitment by leaders to send staff to the trainings and assign a person to be responsible for trainings at the institution. EPHI sent formal invitation letters to selected individuals and was responsible for coordinating logistics to get all participants to the training site.

In July 2018, OSU faculty (a clinical microbiologist/pathologist and infectious disease physicians) trained a cadre of 16 physicians, nurses, and laboratorians, hereinafter referred to as master trainers. The 16 master trainer participants, were selected from hospitals, universities, and EPHI’s National Reference Laboratory, based on experience in collecting specimens for culture, knowledge in promoting excellence in clinical patient care, teaching skills, and leadership and influence in their hospital or institution.

**Fig. 1:**
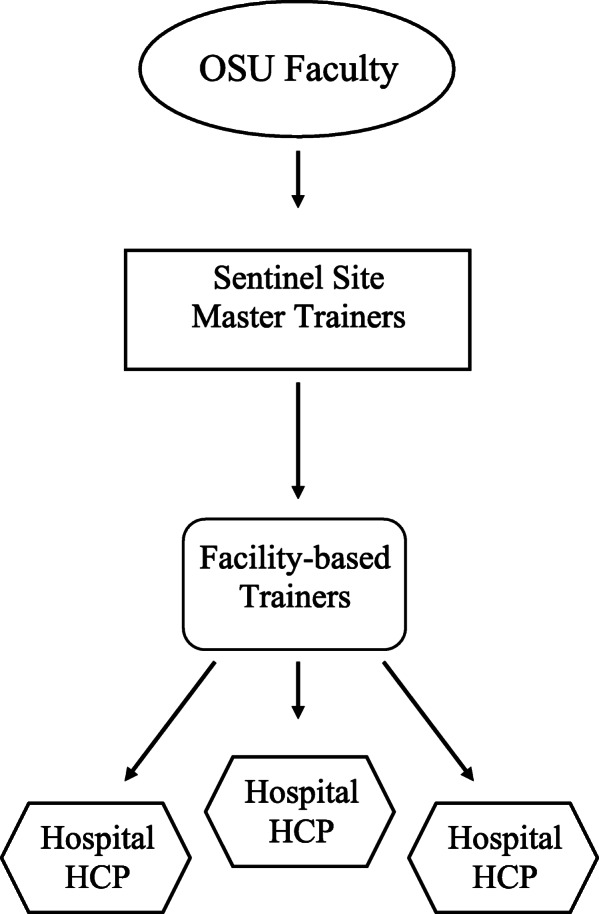
Training-of-Trainers (ToT) Model

The newly developed cadre of master trainers subsequently, and under the guidance of OSU faculty, held two ToTs at the national level in February 2019 and August 2019 where they trained 47 facility-based trainers. These facility-based trainers consisted of HCP (nurses, physicians, and laboratorians) from sites participating in Ethiopia’s AMR Surveillance Network, who would ultimately be responsible for cascading an abridged, in-service version of the training to front-line HCP at their respective institutions (Fig. 1).

### Training procedures

The same course content was delivered to both master trainers and facility-based trainers. At the start of the course, the trainers allowed time for introductions, overview of the training agenda and goals, and presented general class rules and expectations. The training sessions consisted of a didactic introduction followed by demonstration, emphasizing doing, not just knowing; knowledge assessment (pre/post-test); and competency-based evaluation of performance (skill checklist). Printed materials were made available to participants as deemed necessary and appropriate. The OSU-supported training held by the master trainers for facility-based trainers was organized in the format described below, adapted from the CDC ToT model[5].

#### Training preparation

Prior to the start of the course, master trainers gathered all materials, equipment, and supplies for facilitating the training, as listed in the Trainer’s Manual. Master trainers reviewed all content of the Trainer’s Manual so that they had the prior knowledge and background needed before beginning the training for facility-based trainers. The trainers also met with OSU faculty for a half-day preparation session prior to hosting the two-day training.

### Pre‐assessment

**Fig. 2:**
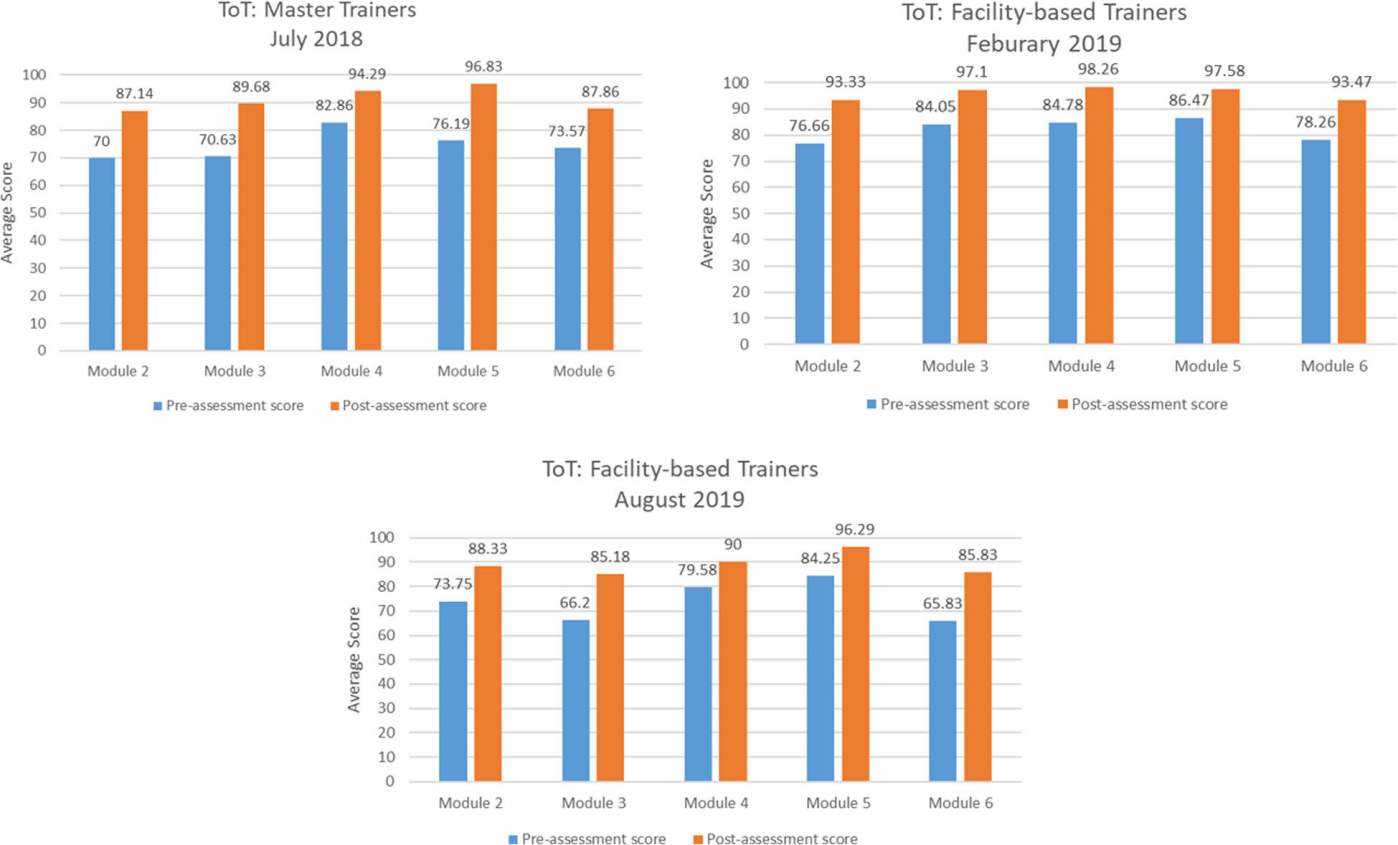
Pre- and Post-assessments for Modules 2 to 6

Master trainers delivered a pre-training questionnaire to determine facility-based participants’ individual and group knowledge on clinical specimen collection. Thus, identification of the healthcare facilities’ needs/gaps occurred in advance and throughout the training. Better understanding of these gaps allowed trainers and OSU faculty to spend more time on certain topics, demonstrate a particular procedure, or emphasize specific points. Average scores among trainees increased after each module training. Pre and post-assessment scores of modules 2 through 6 for the three trainings held from July 2018 to August 2019 are presented in Fig. 2.

### Knowledge delivery and skill modeling

The format of the training was built around skill, practice, and feedback, which provided opportunities for demonstration and practice of each individual’s clinical specimen collection skills. In addition to didactic delivery, master trainers modeled all skills as part of the training modules. At the end of each module, master trainers provided information and instructions to participants for demonstration and teach-back exercises. Depending on the size of the group, participants worked in pairs or threes, following the protocol for each step. Participants were first asked to demonstrate and present to other participants using a “teach back” style. This approach of informal role playing enables strengthening of presentation and teaching skills while maintaining a supportive environment. Participants then provided each other feedback in real-time, in addition to offering comments to improve and further contextualize the materials. Master trainers used participatory and group learning approaches and techniques throughout the activities to help participants acquire the required knowledge, attitudes, and skills. These training activities encouraged the participants to see, analyze, and share their practice and experiences on clinical specimen collection. For example, one person was the specimen collector, one person was the patient, and then the roles were reversed. Additionally, the master trainers engaged participants in creating a plan to outline the sequence of steps that must be taken, or activities that must be performed, for their particular job position to ensure that proper clinical specimen collection can occur and the strategy will succeed.

Participants completed an evaluation at the end of each training day. The evaluation included questions on satisfaction with the training, organization, usefulness of information, format, pace, and knowledge and preparedness of trainers. Participants also rated each master trainer and provided comments to open-ended questions on recommendations for the trainers, the most valuable part of the training, and ways to improve the training. Responses were on a 5-point Likert Scale with 1 = strongly disagree to 5 = strongly agree. Overall, participants were satisfied with the training with average scores for all three trainings ranging from 4.67 to 4.86 (data not shown). Participant agreed that the training was organized and easy to follow (range 4.7 to 4.86), the information was useful (range 4.74 to 5.0), and that the pace was sufficient (range 4.63 to 4.86). Participants frequently commented that the skills demonstration portion in each module was most useful and that the training was practical and easy to understand. Participants also commented that what they liked the most about the trainers were their clear communication skills, knowledge of the topic, and ability to engage the audience. The evaluation data were used to improve subsequent trainings throughout the program.

### Post‐assessment

Master trainers administered a post-assessment questionnaire to determine individual and group-level knowledge on the importance and techniques for clinical specimen collection (see Fig. 2). This allowed for persistent gaps in knowledge to be the focus of potential follow-up support. Follow-up support, while not a direct activity in the project, is important as it is intended to strengthen the transfer of learned strategies or skills so they will be retained and applied effectively.

### Cascade training with mentorship

Following the two ToTs held at the central level by master trainers, newly trained facility-based trainers returned to their facilities to further cascade the materials. While facility trainers were provided the trainers manual, they were also provided copies of the reference manual to disseminate amongst staff for self-learning, reference, and use during the final cascade at the facility level. Methods for training front-line HCP were left to the facility-based trainer to allow flexibility based on time and space resources and participant needs. Options discussed for final cascade training included morning sessions, continuing medical education opportunities, one-hour series over the course of a month, and as part of new staff and medical student orientation. Recommendations were made that facility-based trainers include a component of follow-up and support following the cascade to assess the front-line HCP performance and retention of specimen collection knowledge using the same competency checklists and performance standards provided in the trainer’s manual. During the follow-up, the facility-based trainer could direct feedback to the participant, develop an action plan, and review the implementation of the action plan with the participant, supervisor, and coworkers.

## Discussion

The Clinical Specimen Collection training supports the Ethiopian National AMR Surveillance Plan[7] by providing appropriate clinical specimen collection and emphasizing the importance of proper and timely specimen transport in addition to enhancing diagnostic stewardship. For the sustainable success of such measures, adequate resource allocation and stakeholder engagement is critical from inception onwards.

### Lessons learned and recommendations

The lessons learned from developing and implementing this training are as follows:

#### Training material modifications

Revisions to the material are to be expected; therefore, it must be considered as a living document for the country of Ethiopia. The OSU team made modifications to the manual and other materials as an iterative process with trainees, EPHI, and the CDC. Feedback from master trainers included adapting the course content to the local hospital context, using plain language, translating materials into Amharic, and adding a video as supplemental material. Trainings for facility-based trainers were also facilitated in Amharic; a request made by trainees as implementation in their respective institutions would be conducted in their native language. Furthermore, master trainers used real-world experiences and examples throughout facilitation of the modules; thus, ensuring cultural relevance and appropriateness. Based on the recommendations, the OSU team revised all of the course materials and created a comprehensive training package that included new materials, such as the reference guide and step-by-step instructional guide. Subsequent revisions are planned to be led and managed by EPHI as they continue to use the materials for the recommended refresher trainings.

#### Modes of delivery

The training and reference manuals may be used with the core module PowerPoint slides and assessment tools if time and resources permit. Due to limited training time, most of the ToT was performed using the PowerPoint modules, and participants were provided with the manuals they could readily return to for review or for use during specimen collection procedures. At the facility-level, this curriculum can be administered in a group-based setting or may be used in self-study and individual review with the reference manual alone should limited resources exist. Using a modular approach, the course allows modules to be taught individually (stand-alone) or combined (full or partial course) based on HCP participants’ needs (type of specimen collection training needed) and time availability (1 day, 4 h, or 1 h). Training sessions for HCP may be as short as 30–45 min. The trainer may adapt the training schedule and outline according to the specific needs and time constraints of the audience.

#### Technology

Feedback from training participants recommended providing supplemental video materials; however, videos were not provided due to concerns about accessibility of the internet, unreliable access to video projection equipment, and language issues. For this type of training, videos can be a helpful aid in explaining content, but hands-on demonstrations and peer group discussions may provide more valuable ways to clearly demonstrate knowledge and skills.

#### Sustainability

A frequently stated recommendation from training participants urged institutions to provide periodic refresher trainings (e.g., half-day or shorter 2-hour sessions) for all front-line HCP as circumstances dictate for continuing education. EPHI currently advocates for clinical specimen collection training and mentorship to be made a priority among hospital leadership. Moreover, the training could be integrated into hospital onboarding orientation and/or recognized as professional development activities within hospital settings. Some of the issues around sustainability of this training will depend on individual institutions and facilities. Leadership at these institutions, as well as country stakeholders, will need to take ownership for championing the importance of proper clinical specimen collection. Institutions will have to invest in human capital and provide the necessary training for further professional development among HCP. This will benefit not only the institutions in building capacity for competent HCP, but also enhance the delivery of care and, ultimately, improve patient outcomes.

Careful coordination with ministry stakeholders, such as EPHI, the Ministry of Health, and Ministry of Sciences and Higher Education, will enhance the delivery and uptake of the training curriculum. Ongoing involvement of key stakeholders in Ethiopia, including professional societies, would facilitate the sustained incorporation of the training materials into didactic and professional development courses, which in turn will support more complete adoption of proper collection techniques.

## Limitations of the project

This project was not without limitations. Due to distance and a lack of time, OSU trainers did not observe training implementation in healthcare sites to assess training fidelity, or refinement of materials for the local hospital context. Specimen collection was only simulated in the training and not performed on real patients. Ideally, an assessment of the competencies learned and how that information is transferred into practice is an integral part of educational trainings. In this case, OSU staff gave feedback only during the training-of-trainers. A competency checklist designed for evaluating clinical skills in the healthcare setting was developed and is a part of the training package, but was not used due to time limitations. It is envisaged that the training package could be easily adapted by in-country leads to accommodate revisions, such as translation and other technical refinements, as the context demands. This report only includes the process of training development. In a future report, we will evaluate the outcomes of the training, including the numbers of HCP trained at the facility level, rates of specimen rejection, and rates of contamination.

## Conclusions

Despite limitations, this project demonstrates that development and implementation of a standardized clinical specimen collection training for HCP in a low-resource setting is feasible. The training materials developed can easily be adapted to other settings and languages and allow flexibility in the modes of training delivery and use. Future plans are to conduct a robust evaluation to understand impact of the training curriculum as it has been rolled out in Ethiopia and inform improvements to the training package. The process of developing the materials, as well as how and when to engage local staff and implement roll out, could be very helpful for those who would like to implement similar initiatives. Current plans are to make the materials available to other countries to adapt, implement, and share lessons learned from the roll out. To do this, we are providing a generic, open access suite of training materials via The Ohio State University’s learning management system, CANVAS, online platform: https://www.canvas.net/browse/osu/global-one-health. Through engagement with the aforementioned online resources, other low-middle income countries may be able to establish their own education and training programs on proper specimen collection as well.

## Data Availability

The training modules are available for use at: https://www.canvas.net/browse/osu/global-one-health. We ask that the data be cited accordingly.

## Abbreviations

HCP: Healthcare personnel
LMIC: Low- and middle-income countries
EPHI: Ethiopian Public Health Institute
CDC: Centers for Disease Control and Prevention
OSU: Ohio State University
GOHi: Global One Health initiative
AMR: Antimicrobial resistance
ToT: Training-of-trainers
NCEZID: National Center for Emerging and Zoonotic Infectious Diseases

## References

[1] Shirey C, Perrego K (2015). Standardizing the handling of surgical specimens. AORN J.

[2] Kebede Y, Fonjungo PN, Tibesso G (2016). Improved specimen-referral system and increased access to quality laboratory services in ethiopia: The role of the public-private partnership. J Infect Dis.

[3] Melkie M, Girma A, Tsalla T. The practice of venous blood collection among laboratory and non-laboratory professionals working in ethiopian government hospitals: A comparative study. BMC Health Serv Res. 2014;14:88-6963-14-88. 10.1186/1472-6963-14-88 PMC3943498 24568673

[4] Ibrahim R, Teshal A, Dinku S (2018). Antimicrobial resistance surveillance in ethiopia: Implementation experiences and lessons learned. African Journal of Laboratory Medicine.

[5] Centers for Disease Control and Prevention. Understanding the training of trainers (ToT) model. https://www.cdc.gov/healthyschools/trainingtools.html. Updated 2018.

[6] Bryan RL, Kreuter MW, Brownson RC (2009). Integrating adult learning principles into training for public health practice. Health Promotion Practice.

[7] The Ethiopian Public Health Institute. The surveillance of antimicrobial resistance using public health laboratory-based sentinel sites in Ethiopia, 2016–2020. 2017.

